# Characterization of Hydrocortisone Biometabolites and 18S rRNA Gene in *Chlamydomonas reinhardtii* Cultures

**DOI:** 10.3390/molecules13102416

**Published:** 2008-10-01

**Authors:** Younes Ghasemi, Sara Rasoul-Amini, Mohammad Hossein Morowvat, Mohammad Javad Raee, Mohammad Bagher Ghoshoon, Fatemeh Nouri, Narges Negintaji, Rezvan Parvizi, Seyed Bagher Mosavi-Azam

**Affiliations:** 1 Department of Pharmacognosy and Pharmaceutical Sciences Research Center, Faculty of Pharmacy, Shiraz University of Medical Sciences, Shiraz, Iran; 2 Department of Medicinal Chemistry, and Pharmaceutical Sciences Research Center, Faculty of Pharmacy, Shiraz University of Medical Sciences, Shiraz, Iran

**Keywords:** Biotransformation, *Chlamydomonas reinhardtii*, hydrocortisone, prednisolone, 18S rRNA gene

## Abstract

A unicellular microalga, *Chlamydomonas reinhardtii*, was isolated from rice paddy-field soil and water samples and used in the biotransformation of hydrocortisone (**1**). This strain has not been previously tested for steroid bioconversion. Fermentation was carried out in BG-11 medium supplemented with 0.05% substrate at 25°C for 14 days of incubation. The products obtained were chromatographically purified and characterized using spectroscopic methods. 11β,17β-Dihydroxyandrost-4-en-3-one (**2**), 11β- hydroxyandrost-4-en-3,17-dione (**3**), 11β,17α,20β,21-tetrahydroxypregn-4-en-3-one (**4**) and prednisolone (**5**) were the main products of the bioconversion. The observed bioreaction features were the side chain degradation of the substrate to give compounds **2** and **3** and the 20-ketone reduction and 1,2-dehydrogenation affording compounds **4** and **5**, respectively. A time course study showed the accumulation of product **2** from the second day of the fermentation and of compounds **3**, **4** and **5** from the third day. All the metabolites reached their maximum concentration in seven days. Microalgal 18S rRNA gene was also amplified by PCR. PCR products were sequenced to confirm their authenticity as 18S rRNA gene of microalgae. The result of PCR blasted with other sequenced microalgae in NCBI showed 100% homology to the 18S small subunit rRNA of two *Chlamydomonas reinhardtii* spp.

## Introduction

Microbiological conversions of steroids, specially enzymatic 1,2-dehydrogenation of pregnane derivatives, have received considerable attention because dehydrogenated corticosteroid derivatives are usually more effective than their precursors in clinical application [1]. The use of biocatalysts is preferred to chemical transformation processes for the preparation of single isomers of a product. The ability of microalgae to transform exogenous steroids has been described in several of our previous publications [2,3,4]. The topic has also been the subject of a recent review [5]. They are easily and rapidly cultured in inexpensive media containing simple salts, which decrease the probability of contamination by other microorganisms [4]. With regards to the substrate used in our experiments, the use of fungi and bacteria for 1,2-dehydrogenation of hydrocortisone have been reported by Adham’s group [6]. They examined thirty-nine of a wide range of microorganisms, 15 fungi and 24 bacteria, for their ability to ∆^1^-dehydrogenate the A ring of hydrocortisone for the production of prednisolone. Among of them, *Pseudomonas fluorescens* showed the greatest bioconversion efficiency [6].

*Chlamydomonas reinhardtii* is a motile single-celled green alga about 10 micrometers in diameter that swims with two flagella. These algae are distributed all over the world and occur in soil and fresh water [7]. Preliminary taxonomical studies showed that *Chlamydomonas reinhardtii* was quite common in the fresh water and paddy fields of Fars province in the south of Iran. Other microalgae like *Oocystis*, *Chlorella*, *Scenedesmus* and some unicellular and filamentous cyanobacteria are also found in the same locations [4]. Several studies have used *Chlamydomonas* spp. for biotransformations or biosorbtions of organic compounds. In one study, a *Chlamydomonas* species was shown to transform naphthalene into 1-naphthol [8]. In another study, a *Chlamydomonas* sp. was investigated for its ability to transform lindane, naphthalene and phenol [9]. The halogenation of 4-chloro-3,5- dinitrobenzoic acid by a *Chlamydomonas* sp. gave 2-hydroxymuconic semialdehyde [10]. Liebe and Fock [11] showed that *Chlamydomonas reinhardtii* was able to remove some of the toxic *iso*-octane- extracted polycyclic aromatic hydrocarbons (PAHs) from diesel particulate exhaust. The PAH derivatives were consumed as nutrients. In other investigations, pyrene was removed by biosorbtion, bioaccumulation and biotransformation in *Chlamydomonas* spp. [12]. Wang’s group demonstrated the biodegradation and biosorbtion of triazophos, by *Chlamydomonas reinhardtii* [13] and Hirooka and co-workers described the removal of 2-amino-4-nitrophenol [14].

In continuation of our work on the bioconversion of steroids by *Nostoc muscorum* [2], *Choroococcus dispersus* [15], *Fischerella ambigua* [16] and *Nostoc ellipsosporum* [3], the biotransformation of hydrocortisone as an exogenous steroid was carried out using a locally isolated strain of the unicellular microalga, *Chlamydomonas reinhardtii*. Until now, *Chlamydomonas reinhardtii* has not been examined in the context of the transformation of steroids. The aim of this

study was to examine the ability of this organism to convert of hydrocortisone to prednisolone. In addition, the partial sequence of the 18S rRNA gene of the *Chlamydomonas reinhardtii* was identified.

## Results and Discussion

### Identification of the algal strain

The strain was recognized by morphological characterization and assigned according to 18S rRNA gene sequence. The classification of the isolate alga was performed by Microalgal Culture Collection of Shiraz University of Medical Sciences, Faculty of Pharmacy, Shiraz, Iran, as a strain of *Chlamydomonas reinhardtii* MCCS 002.

### Partial sequence of the 18S rRNA

The partial sequence of the 18S rRNA sequence of the *Chlamydomonas reinhardtii* MCCS 002 is as follows:

5′attgatcaagaacgaaagttgggggctcgaagacgattagataccgtcgtagtctcaaccataaacgatgccgactagggatt ggcagatgttcttttgatgactctgccagcaccttatgagaaatcaaagtttttgggttccggggggagtatggtcgcaaggctgaa acttaaaggaattgacggaagggcaccaccaggcgtggagcctgcggcttaatttgactcaacacggggaaacttaccaggtc cagacacgggaaggattgacagattgagagctctttcttgattctgtgggtggtggtgcatggccgttcttagttggtgggttgcctt gtcaggttgattccggtaacgaacgagacctcagcctgctaaatagtcagcatcgcacctgcggtgcgccgacttcttagaggg actattggcgtttagccaatggaagtatgaggcgataacaggtctgtgatgcccttagatgttctgggccgcacgcgcgctacact gacgcgaccaacgagcctatccttggccgagaggcccgggtaatc 3′

The DNA sequence of *Chlamydomonas reinhardtii* strain MCCS 002 was recorded in the NCBI under the accession number EF682842. The result of PCR BLAST comparison with other sequenced microalgae in NCBI showed 100% homology to the 18S small subunit rRNA of two strains of *Chlamydomonas reinhardtii*. Other sequences showed 96-99% identity and most belonged to the genera of *Tetrabaena*, *Chlamydomonad*, *Volvox*, *Tetraspora*, *Chloromonas* and *Chlorogonium*.

### Biotransformation of hydrocortisone *(**1**)*

**Figure 1:**
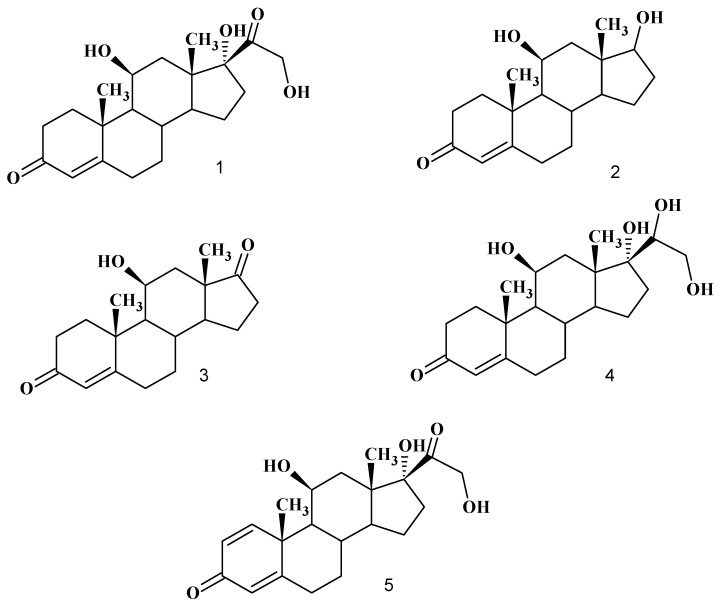
Chemical structure of substrate hydrocortisone (**1**) and the biotransformed products 11β,17β-dihydroxyandrost-4-en-3-one (**2**), 11β-hydroxyandrost-4-en-3,17-dione (**3**), 11β,17α,20β,21-tetrahydroxypregn-4-en-3-one (**4**) and prednisolone (**5**).

The crude extract obtained from 14 day incubation of *Chlamydomonas reinhardtii* MCCS 002 in the presence of hydrocortisone (**1**) produced two androstane compounds: 11β,17β-dihydroxyandrost-4- en-3-one (**2**), 11β-hydroxyandrost-4-en-3,17-dione (**3**) and two pregnane compounds: 11β,17α,20β, 21-tetrahydroxypregn-4-en-3-one (**4**) and prednisolone (11β,17α,21-trihydroxypregn-1,4-diene-3,20- dione, **5**) in addition to the untransformed substrate **1** (Figure 1).

For the time course study the production of **2-5** as a function of incubation time was monitored by thin layer chromatography (TLC). Compounds **2** and **3** both were less polar (R_f_: 0.6 and 0.7 respectively) than the substrate (R_f_: 0.45) and the other metabolites **4** and **5** were much more polar (R_f_: 0.18 and 0.4, respectively). The starting material **1** (1 mg·mL^-1^) was transformed into the products within seven days. According to the TLC profile, metabolite **2** accumulated in the broth from the second day of incubation and reached its maximum concentrations within seven days, while metabolites **3**, **4** and **5** appeared in the fermentation broth three days after adding the starting material. *Chlamydomonas reinhardtii* was also seen to convert hydrocortisone at different concentrations ranging between 0.5 to 2.5 mg·mL^-1^. At concentrations above this latter limit hydrocortisone was not converted to any metabolite. Based on the TLC profiles, the best substrate concentration was 1 mg·mL^-1^.

### Product characterization

The mass spectra of compounds **2** and **3** showed the corresponding molecular ions at *m/z* 304 and 302, respectively, suggesting reductions by 58 and 60 mass units, respectively, compared to hydrocortisone (*m/z* 362). The IR spectra indicated the existence of at least one hydroxyl group in both compounds. Furthermore, in compound **2**, the IR spectra showed a single carbonyl group at 1657 cm^-1^, which was conjugated with the C4-C5 double bond. The resonances at δ 3.82 and 4.37 in the ^1^H-NMR spectrum clearly showed the existence of two hydroxyl groups. The reported chemical shift for H-11 in hydrocortisone and other 11-hydroxylated steroids is in the δ 4.3–4.4 range [3], so the resonance at δ 3.82 was attributed to a C-17 CH–OH moiety. These data were supported by the ^13^C-NMR data, which showed a downfield resonance at δ 84.6 for the C-17 CH–OH. In compound **3**, the IR spectrum showed two absorptions at 1663 and 1734 cm^-1^, which confirmed the existence of two carbonyl groups at C-3 and C-17, respectively. These IR data have also been supported by the corresponding ^13^C-NMR spectrum, where two signals at δ 199.9 and 219.8 were attributable to C-3 and C-17, respectively.

The mass spectrum of metabolite **4** showed the molecular ion at *m/z* 364, which compared to that of hydrocortisone (*m/z* 362) indicated the addition of two mass units. It can be imagined that either a carbonyl group or a double bond in the substrate have been reduced. The IR spectrum showed only one carbonyl group at 1661 cm^-1^, suggesting that the conjugated ketone in C-3 position has not been altered and that the reduction had taken place at C-20. An additional multiplet resonance at δ 3.70 in the ^1^H-NMR spectrum, compared to that of the substrate, confirmed the proposed structure of metabolite **4**. In addition, the other notable feature observed was the stereospecificity of the reduction of the C-20 ketone group. The configuration of the C-20 hydroxyl group was determined mainly by comparison of its melting point with that of compounds having α-hydroxyl and β-hydroxyl groups at C-20 [3]. The melting point value of metabolite **4** was similar to that reported for the compound with a β-hydroxyl group at the C-20 position.

The mass spectrum of metabolite **5** showed the molecular ion at *m/z* 360, which indicated the decrease of two units as compared to that of hydrocortisone. This can occur by the dehydrogenation of a C-C bond in the substrate. The ^1^H-NMR spectrum of metabolite **5** was virtually identical to that of hydrocortisone (**1**) except for a further shift downfield of the 4-H signal to δ 5.96 from 5.55 in hydrocortisone, and for the presence downfield of signals for 1-H (δ 7.2) and 2-H (δ 6.2). This position of the 1-H and 2-H proton signals is characteristic of 1,2-dehydrogenation in ring A. IR peaks at 1600 cm^-1^ (3-C=O), 1658 and 1624 cm^-1^ (1,4-diene) all supported the structural assignment. The ^13^C-NMR chemical shifts of compound **5** exhibited signals at δ 213 and 185.2, corresponding to two carbonyl groups at C-20 and C-3, respectively [3,17].

## Conclusions

In summary, as far as we know the transformation of hydrocortisone by *Chlamydomonas reinhardtii* has never been reported before. Hydrocortisone is one of the most useful intermediates for production of some valuable pharmaceutically steroid compounds and has been used in many studies as a substrate in biotransformation experiments [18]. Earlier studies have clearly demonstrated that bacteria and fungi can metabolize hydrocortisone. 1,2-Double bond formation using *Cylindrocarpon radicicola*, *Streptomyces lavendulae*, *Fusarium caucasicum*, *Fusarium solani* and *Septomyxa affinis* and 1,2-dehydrogenation of hydrocortisone to prednisolone by *Arthrobacter simplex*, *Bacillus sphaericus* and *Bacterium cyclooxydans* have already been applied in industrial production processes [16]. In our previous research on hydrocortisone bioconversion using cyanobacterial strains, it was reported that *Nostoc muscorum* [2], *Fischerella ambigua* [16], and *Nostoc* *ellipsosporum* [3] converted the substrate into some pregnane- and androstane-derived products. The most notable result in this study has been the 1,2-dehydrogenation of hydrocortisone to prednisolone (**5**), which is reported here for the first time. The use of this microbial bioconversion would reduced the number of steps normally required to prepare prednisolone and could consequently reduce its production cost [1]. In this study, we have also shown that *Chlamydomonas reinhardtii* can achieve steroidal side-chain cleavage in a pregnane-based steroid. The nature of the enzyme responsible for the side-chain cleavage in this microorganism is not clearly known, but it could be similar to the one which catalyses the conversion of cholesterol to pregnenolone in mammals and it may be a cytochrome P450 [14]. As these results show that the *Chlamydomonas reinhardtii* MCCS 002 may be considered a useful biocatalyst for 1,2- dehydrogenation of steroid substrates. It has a potential for site- and regioselective bioconversion of hydrocortisone and probably other androstane like steroids.

## Experimental

### General

Melting points (m.p) were determined on a Reichert-Jung hot stage melting point apparatus. Optical rotations were measured in 1 dm cells on a Perkin-Elmer 142 automatic spectropolarimeter. ^1^H- and ^13^C-NMR spectra were recorded in CDCl_3_ on a Bruker DRX-500 Avance NMR spectrometer, with tetramethylsilane (TMS) as internal standard. Chemical shifts (δ) are given in ppm relative to TMS. Coupling constants (*J*) are given in Hertz (Hz). Infrared (IR) spectra were recorded on a Magna-IR 550 Nicolet FTIR spectrometer. Mass spectra (MS) were obtained with a Hewlett-Packard 6890 instrument by electron impact (EI) at 70 eV. Thin layer chromatography (TLC) and preparative TLC were performed on 0.25 and 0.5 mm thick layers of silica gel G (Kieselgel 60 HF_254+366_, Merck), respectively. The layers were prepared on glass plates and activated at 105°C for 1 hour before use. Chromatography was performed with acetone/hexane (1:1, v/v) and visualized by spraying the plates with a mixture of methanol/sulfuric acid (6:1, v/v) and heating in an oven at 100°C for 3 min until the colors developed. The compounds were also visualized under UV lamp (Strstedt-Gruppe HP-UVIS) at 254 nm.

### Chemicals

Hydrocortisone, purchased from Pharmacia and Upjohn S.A. (Guyancourt, USA), was kindly donated by Aburaihan Pharmaceutical Co. (Tehran, Iran). Other reagents and solvents were from Merck.

### Collection, preservation and identification of the alga

*Chlamydomonas reinhardtii* was isolated from soil samples collected from paddy fields in Shiraz, located in the southern part of Iran (Fars province), from April to December 2004 during a screening program. Primary culturing was done in BG-11 medium [19]. After colonization, pure cultures of the living specimens were prepared by subculturing with agar plate method in BG-11 medium [20]. The preserved specimens were prepared and the living specimens were incubated in 50 mL-conical flasks, under unlimited carbon dioxide conditions (using CO_2_ enrichment condition). Constant illumination was used at 60 uE·m^-1^·s^-1^ intensity with white fluorescent lamps. The temperature was 25±2° C. The identification was done using standard manuals [21,22]. Physiological analyses including growth rate measurement, pigment analysis and the effect of illumination have been done using the methods described by Soltani *et al*. [23].

### Incubation conditions

The fermentation experiments were conducted in twenty 500-mL conical flasks, each containing BG-11 liquid medium (100 mL). Inocula from the fresh culture of *Chlamydomonas reinhardtii* was used at a final cell density of approximately 2.7-3 × 10^6^ cells mL^-1^ and illuminated continuously with fluorescent lamps at 60 uE·m^-1^·s^-1^ intensity, and incubated at a temperature of 25±2 °C with shaking at 80 rpm for seven days. Hydrocortisone (1 g) was dissolved in ethanol (20 mL) and one milliliter of the ethanol solution was added to each 500-mL conical flask (final concentration of the substrate was 0.05% in each flask). Incubation was continued for another 14 days at the same conditions and the control was similarly processed without the microorganism. We also examined the optimum substrate concentration. The amount of the substrate was varied from 0.025 to 0.2 g 100 mL^-1^ in 0.25 g steps. The results were checked by TLC analyses. Cell density (number of cell mL^-1^) was determined by both turbidity (optical density) and direct counting, using a light microscope with a 0.1 mm deep counting chamber (Neubauer haemocytomer). Correlation between these two methods was analyzed reaching to certainty [24].

### Time course study and the effect of substrate concentration

For a time course study, *Chlamydomonas reinhardtii* was transferred into a 500-mL Erlenmeyer flask containing BG-11 broth (100 mL) supplemented with hydrocortisone (50 mg). Then the incubation was carried out for seven days at the condition described above. Sampling was carried out every 24 hours. The amount of the substrate varied from 0.025 to 0.2 g 100 mL^-1^ in 0.25 g increments.

### Products isolation and analysis

At the end of incubation, the content of the flasks was extracted with three volumes of chloroform. The extract was dried over anhydrous sodium sulfate and evaporated under reduced pressure. The residue was loaded on preparative TLC and fractionated with chloroform/acetone (1:1 v/v) solvent system and then the metabolite was crystallized in ethanol. Purified metabolites were identified by melting point and spectral data (^13^C-NMR, ^1^H-NMR, FTIR, and MS). The yields of the bioproducts were calculated according to the percentage of dry weight of each compound.

*11β, 17β-Dihydroxyandrost-4-en-3-one* (**2**). Metabolite **2** was crystallized from methanol; yield 10%; m.p. 240-242°C, [α]_D_ +164° (MeOH) (lit. [15]: m.p. 241-243°C, [α]_D_ +142°); IR ν_max_ (KBr, cm^-1^) 3433, 2976, 1657; MS (EI) *m/z* (%) 304 (78) (M^+^, C_19_H_28_O_3_), 303 (20), 261 (100), 235 (55), 188 (20), 123 (60), 109 (50), 82 (85); ^1^H-NMR δ 1.11 (3H, s, H-18), 1.47 (3H, s, H-19), 3.82 (1H, m, H-17), 4.37 (1H, m, H-11), 5.77 (1H, s, H-4); ^13^C-NMR δ 199.9 (C_3_), 171.4 (C_5_), 122.7 (C_4_), 84.6 (C_17_), 67.1 (C_11_), 55.4 (C_9_), 49.4 (C_14_), 46.2 (C_12_), 43.2 (C_13_), 38.4 (C_1_), 35.6 (C_10_), 35.5 (C_16_), 34.3 (C_2_), 32.6 (C_6_), 32.2 (C_7_), 32.6 (C_6_), 31.1 (C_8_), 25.8 (C_15_), 21.3 (C_19_), 15.3 (C_18_); R_f_ 0.6 (chloroform/acetone; 1:1 v/v).

*11β-Hydroxyandrost-4-en-3,17-dione* (**3**). Metabolite **3** was also crystallized from methanol; yield 14%; m.p. 196-199°C, [α]_D_ +226°(MeOH); (lit. [15]: m.p. 197-198°C, [α]_D_ (CHCl_3_) +225°); IR ν_max_ (KBr, cm^-1^) 3522, 1734, 1663; MS (EI) *m/z* (%) 302 (100) (M^+^,C_19_H_26_O_3_), 286 (32), 227 (41), 189 (64), 149 (40), 123 (80); 91 (80), 75 (60); ^1^H-NMR δ 1.20 (3H, s, H-18), 1.52 (3H, s, H-19) , 4.52 (1H, s, H-11), 5.74 (1H, s, H-4); ^13^C-NMR δ 219.8 (C_17_), 199.9 (C_3_), 171.7 (C_5_), 122.8 (C_4_), 68.6 (C_11_), 57.1 (C_9_), 52.8 (C_14_), 47.1 (C_13_), 39.7 (C_10_), 41.4 (C_12_), 31.4 (C_8_), 35.7 (C_1_), 35.4 (C_16_), 34.2 (C_2_), 32.2 (C_6_), 31.9(C_7_), 22.1 (C_15_), 21.5 (C_19_), 16.3 (C_18_); R_f_ 0.7 (chloroform/acetone; 1:1 v/v).

*11β,17α,20β,21-Tetrahydroxypregn-4-en-3-one* (**4**). Metabolite **4** was crystallized from methanol; yield 26%; m.p.: 132-134°C, [α]_D_ +91° (MeOH); (lit. [2]: m.p.: 133-135°C, [α]_D_ +85°); IR ν_max_ (KBr, cm^-1^) 3536, 2910, 1661; MS (EI) *m/z* (%) 364 (18) (M^+^,C_21_H_32_O_5_), 346 (19), 331 (8), 315 (56), 303 (46), 285 (100), 267 (31), 227 (64), 148 (38), 124 (40), 91 (82), 79 (55); ^1^H-NMR δ 1.1 (3H, s, H- 18), 1.43 (3H, s, H-19), 3.63 (2H, dd, J=18.2 Hz, J=4.9 Hz, H-21), 3.70 (1H, m, H-20 ), 4.37 (1H, s, H-11), 5.66 (1H, s, H-4); ^13^C-NMR δ 199.8 (C_3_), 172.7 (C_5_), 122.1 (C_4_), 84.2 (C_17_), 74.3 (C_20_), 68.2 (C_11_), 64.2 (C_21_), 55.3 (C_9_), 50.5 (C_14_), 46.6 (C_13_), 41.3 (C_10_), 39.4 (C_12_), 33.7 (C_1_), 33.2 (C_2_), 32.8 (C_16_), 32.1 (C_6_), 29.7 (C_7_), 29.3 (C_8_), 23.6 (C_15_), 20.8 (C_19_), 17.7 (C_18_); R_f_: 0.18 (chloroform/acetone; 1:1 v/v).

*Prednisolone* (**5**). Metabolite **5** was crystallized from methanol; yield 18%; m.p. 233-235°C, [α]_D_ +100° (dioxane); IR ν_max_ (KBr, cm^-1^) 3050, 2980, 1658, 1624, 1600 cm^-1^; MS (EI) *m/z* (%) 360 (7) (M^+^, C_21_H_28_O_5_), 300 (19), 122 (100), 91 (14), 148 (38), 124 (40), 74 (35) ,55 (45); ^1^H-NMR δ 0.9 (3H, s, H- 18), 1.3 (3H, s, H-19), 4.42 (1H, m, H-11), 4.60 (1H, dd, H-21), 5.96 (1H, s, H-4), 6.20 (1H, d, H-2), 7.20 (1H, d, H-1); ^13^C-NMR δ 213 (C_20_), 185.2 (C_3_), 170.4 (C_5_), 156.8 (C_1_), 127 (C_2_), 121.8 (C_4_), 88.6 (C_17_), 68.8 (C_11_), 66.4 (C_21_), 55.4 (C_9_), 51.2 (C_13_), 55.4 (C_9_), 47.2 (C_12_), 44.2 (C_12_), 38.8 (C_10_), 34.2 (C_8_), 33.6 (C_16_), 31.8 (C_6_), 31.2 (C_7_), 23.9 (C_15_), 21 (C_19_), 17.2 (C_18_); R_f_ 0.4 (chloroform/acetone; 1:1 v/v).

### 18S ribosomal RNA sequencing

For this purpose, DNA content was first extracted from the *Chlamydomonas reinhardtii* and then PCR was applied using two set primers. The sequences were amplified using the primers 5′-GTCAGAGGTGAAATTCTTGGATTTA-3′ as forward and 5′-AGGGCAGGGACGTAATCAACG-3′ as reverse, which amplify a ~600-bp region of the 18S rRNA gene. The applied PCR condition has been described by Nubel *et al*. [25]. PCR products were electrophoresed in a 1% (w/v) agarose gel using TBE buffer containing 1μg/mL ethidium bromide. A single ~600-bp band of DNA was cut and extracted from the gel using the Core Bio Gel Extraction Kit. The sequence was determined by the CinnaGen Company with the primers. Sequence similarity searches were done on the NCBI databases with BLAST [26] and the GeneDoc [27] software package.
